# Tracheobronchomegaly (Mounier-Kuhn syndrome) and Bronchiectasis as rare manifestations of Homocystinuria

**DOI:** 10.1016/j.rmcr.2023.101808

**Published:** 2023-01-04

**Authors:** Aasir M. Suliman, Mohamed A. Alamin, Maha M. Hamza

**Affiliations:** Pulmonology Department, Hamad General Hospital, Hamad Medical Corporation, Doha, Qatar

**Keywords:** Homocystinuria, Bronchiectasis, Tracheobronchomegaly, Mounier-Kuhn syndrome, Fibrillin degeneration

## Abstract

Homocystinuria (HCU) is a rare autosomal recessive inherited disorder usually diagnosed in childhood. It is characterized by a deficiency of the enzyme that converts homocysteine to cystathionine. The accumulation of homocysteine leads to abnormalities in the ocular, skeletal, cardiovascular, and central nervous systems. HCU shares several clinical features with Marfan syndrome; however, respiratory system involvement in HCU is uncommon and rarely reported. Bronchiectasis has been previously reported in a few cases of HCU, and it was attributed mainly to fibrillin deficiency. This case describes a young girl diagnosed with classical HCU since childhood who presented with a chronic productive cough and was initially misdiagnosed as bronchial Asthma. However, upon further evaluation, she was eventually diagnosed with tracheobronchomegaly (TBM), or Mounier-Kuhn Syndrome, and bronchiectasis based on the computed tomography (CT) scan of chest findings. To our knowledge, this is the first reported case of TBM and bronchiectasis in HCU. We believe that fibrillin degeneration may be the key to understanding this unusual association in HCU.

## 1 Introduction

Classic homocystinuria (HCU) is an autosomal recessive disorder and is the most common inborn error of methionine metabolism due to cystathionineß-synthase (CBS) deficiency [1]. Biochemically, it is characterized by abnormal accumulation of homocysteine in the blood and urine. Clinically, it classically manifests in a combination of inferior lens subluxation, myopia, intellectual disability, marfanoid habitus, and hypercoagulability [2]. Of note, respiratory manifestations of HCU are uncommon and rarely described. Herein, we report unusual respiratory complications of HCU in the form of bilateral Bronchiectasis and Tracheobronchomegaly (TBM) that were initially misdiagnosed as Bronchial Asthma. This report aims to highlight the correlation and raise awareness of these unusual associations in HCU.

## 2 Case presentation

A 30-year-old female who's diagnosed with classical HCU in early childhood based on clinical and biochemical findings. She has a marfanoid habitus, bilateral lens subluxation with a history of left eye lensectomy and intraocular lens implant, and mild mental retardation. In addition to a blood methionine level of 1010 μmol/L and homocysteine level of 180 μmol/. She has been on regular follow-ups at a specialized metabolic clinic since childhood but had a poor diet and medications compliance. Her family history is remarkable for classical HCU in her younger brother; however, no family history of Bronchiectasis.

The patient has had a chronic cough with multiple episodes of wheezes and lower respiratory tract infections for several months. She was initially evaluated at a general medicine clinic with multiple chest x-rays (CXR) and several failed attempts of spirometry as she couldn't do it optimally. Subsequently, she was treated for possible Bronchial Asthma with a trial of inhalers (fluticasone/formoterol with as-needed salbutamol) and referred to a Pulmonology clinic. Eventually, she was evaluated at a Pulmonology clinic, and at that time, she had stopped taking her inhalers as they were ineffective. Clinically, she complained of chronic productive cough with thick greenish sputum, which she had difficulty clearing out, associated with occasional wheezes and mild exertional shortness of breath. She had no history of fever, night sweats, weight loss, hemoptysis, or features of allergic rhinitis, and no symptoms of gastroesophageal reflux disease (GERD). She is a lifetime non-smoker with good social support. She was comfortable and maintained 100% oxygen saturation on room air on examination. She had no clubbing or cyanosis, and her chest examination was remarkable for mild right basal coarse crackles.

**Fig. 1:**
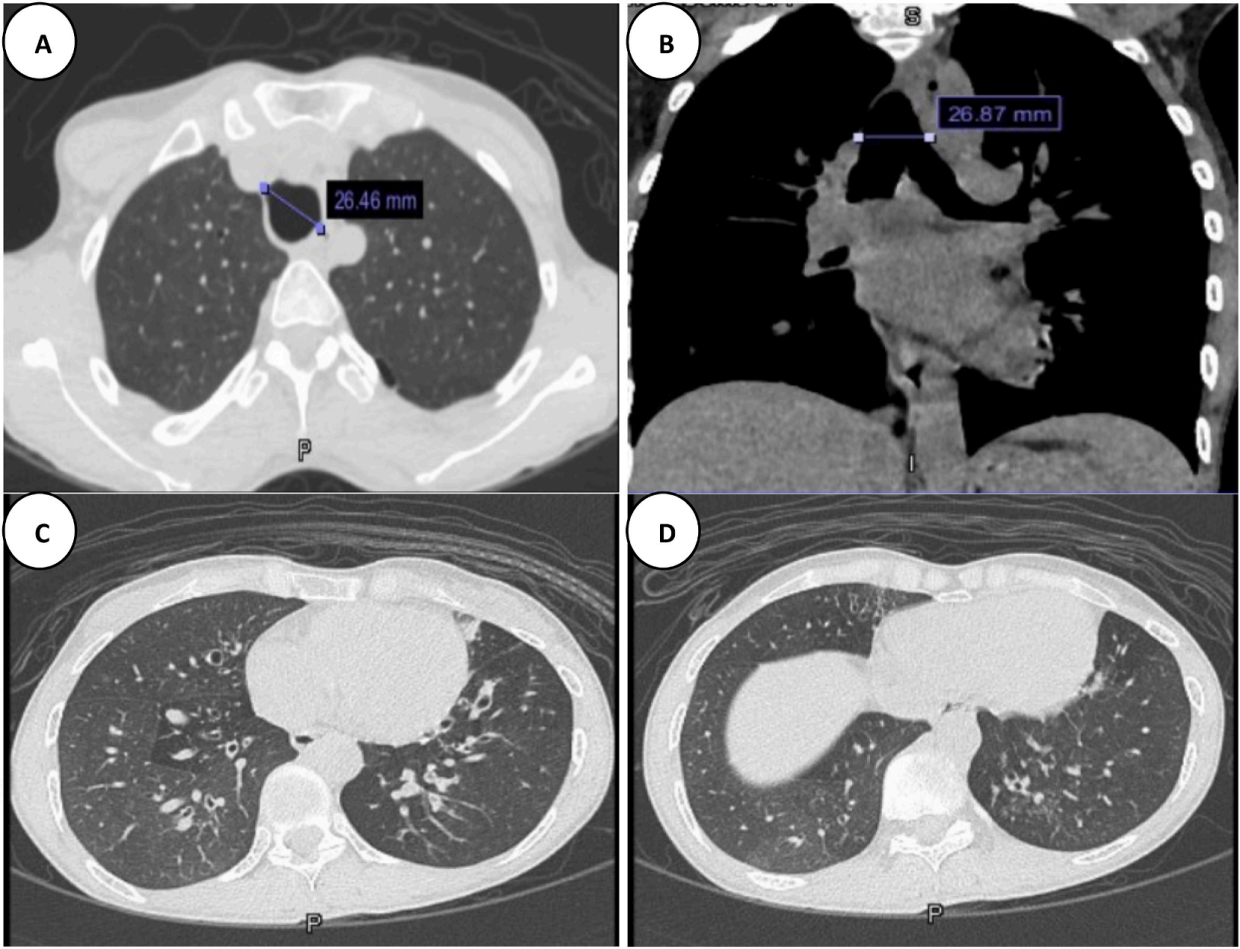
**(A**–**D):** Selected axial and coronal high-resolution CT images show marked dilatation of the trachea (arrow in A & B), dilatation of the main right and left bronchus (B), minimal paraseptal emphysema (A), and bilateral bronchiectasis (C & D).

A computed tomography (CT) scan of the chest revealed abnormally dilated trachea and both main bronchi, along with bronchiectasis over both lung fields. In addition to a single minimal emphysematous lesion. Otherwise, the rest of the lung fields were unremarkable. (Fig. 1). In light of these radiological findings, the diagnosis of Mounier-Kuhn Syndrome was made, and bronchiectasis work-up was initiated to rule out other causes. Blood tests, including complete blood count with differential, immunoglobulins level, autoimmune panel, total IgE level, and Aspergillus fumigatus specific IgE, were all within normal limits. In addition, sputum culture showed moderate growth of sensitive Haemophilus Influenzae; otherwise, negative Mycobacterial and fungal cultures. At this point, the patient was diagnosed with TBM (Mounier-Kuhn Syndrome) and bronchiectasis secondary to HCU. She was started on a multidisciplinary management approach, including dietitian support (with the collaboration of the metabolic team), chest physiotherapy, sputum clearing techniques, appropriate vaccinations, and regular follow-up.

## 3 Discussion

Classical HCU is a rare inherited disorder caused by cystathionine beta-synthase deficiency. The reported incidence of HCU varies between 1: 50,000 to 1:200,000, with the highest rate being in Qatar (1:1800) due to the high rate of consanguinity [3]. The clinical manifestations of HCU have a broad spectrum of severity, from asymptomatic individuals to those with a severe multi-system disease, with a wide range of ages at presentation. Patients present at various timelines if not treated with ocular, neurological, skeletal, and vascular abnormalities [4]. Classical HCU has several phenotypical features in common with Marfan syndrome, including dislocation of the lens; a tall, thin build with long limbs; arachnodactyly; and a pectus deformity of the chest. Pulmonary manifestations of HCU are generally rare; they have been reported in the form of primary spontaneous pneumothorax, massive pulmonary thromboembolism, and restrictive abnormalities due to scoliosis [5]. In addition to bronchiectasis, it has been reported in a few countable instances and postulated to be due to fibrillin degeneration [1,5,6].

TBM, or Mounier-Kuhn Syndrome, is a rare disease characterized by distinct tracheobronchial dilatation with impaired mucociliary clearance that makes affected patients prone to recurrent infections and may eventually progress to bronchiectasis. The underlying pathogenesis of TBM is not clear to date but is postulated due to the atrophy of the muscular and elastic tissues in the tracheobronchial tree [7]. It can be associated with other connective tissue disorders such as Ehlers-Danlos syndrome, cutis laxa, and Marfan syndrome. The diagnosis is based on the characteristic CT chest scan findings; in women, the transverse and sagittal tracheal diameter must, by definition, be greater than 21 and 23 mm, respectively [8]. The clinical presentation varies widely, from asymptomatic patients to recurrent chest infections, wheezing, and exertional dyspnea frequently interpreted as caused by obstructive airway disease, similar to our patient [9]. The treatment is mainly supportive, aiming at preventing and treating infections with designated vaccinations and antibiotics, clearing secretions with mucolytics, and chest physiotherapy. Surgical intervention and lung transplant are reserved for advanced stages of the disease [10].

To our knowledge, this is the first case that describes concurrent TBM and bronchiectasis in HCU. The association of TBM and HCU with marfanoid features in the literature suggests a common underlying defect in fibrillin and elastic tissue. Fibrillin is an essential extracellular matrix glycoprotein forming the elastic fibers found in connective tissue, and it is rich in cysteine, an end product of homocysteine [11]. In HCU, decreased cystathionine and cysteine are associated with apoptosis, oxidative stress, and alterations of fibrillin [4]. As a result, the degenerated fibrillin in HCU may play a role in the structural skeletal and tracheobronchial changes in the marfanoid habitus and TBM, respectively. In our case, we believe the etiology of bronchiectasis is mostly due to TBM and its predisposition to recurrent lower respiratory tract infections. However, as elaborated in the previous reports, the effect of fibrillin degeneration on the small airways' elastic tissue may also be a contributing factor.

Most patients with classical HCU have no clinical complications at birth; however, if not treated, these complications can develop and are mostly irreversible. Although rare, respiratory complications of HCU, including TBM and bronchiectasis, may be attributed to fibrillin degeneration. These respiratory complications can induce substantial morbidity and potentially increase mortality. Thus, this report suggests there is a need for a high suspicion index in HCU patients presenting with respiratory symptoms to allow for early recognition and treatment. Further studies into approaches to prevent respiratory complications are needed, but improved recognition of the respiratory complications of HCU is necessary before this research is likely to begin.

## Declaration of competing interest

The authors declare that they have no known competing financial interests or personal relationships that could have appeared to influence the work reported in this paper.
